# Role of antimicrobials in the treatment of adult patients presenting to the emergency department with acute gastroenteritis - A mini review

**DOI:** 10.12669/pjms.332.11851

**Published:** 2017

**Authors:** Omar Abbas Ahmed Malik

**Affiliations:** 1 Dr. Omar Abbas Ahmed Malik, Student. Dow Medical College, Dow University of Health Sciences, Karachi, Pakistan

**Keywords:** Anti-bacterial agents, Drug therapy, Diarrhea, Dysentery, Gastroenteritis, Review

## Abstract

**Background & Objective:**

Acute gastroenteritis is generally considered a self-limiting illness that does not require the use of antibiotics. However, many emergency departments in the country frequently prescribe antibiotics to patients presenting with diarrhoea. This review attempts to determine whether this practice is reasonable. Our objective was to determine the role of antimicrobials in the empiric management of acute gastroenteritis

**Methods:**

The online data base “PubMed”, as well as the World Wide Web, were searched for relevant articles (RCTs, Reviews, Prospective studies, etc.) with key words such as “gastroenteritis AND antibiotics”, “Management AND gastroenteritis”, “Treatment AND diarrhoea” etc. and covered the years 1960-2016. Fifty articles were studied, of which 43 were chosen on the basis of relevance for qualitative assessment.

**Results:**

The articles reviewed for this paper suggest that antimicrobial therapy is not appropriate for the majority of cases of (uncomplicated) gastroenteritis, as risks (antibiotic-associated diarrhoea, hypersensitivity reactions, etc.) outweigh benefits. However, there are instances where antibiotics are clearly indicated. Further, it is noted that there have not been any recent trials to clarify the role of antimicrobials in adult diarrhoeal illness.

**Conclusions:**

The focus in management of patients presenting with diarrhoea in the Emergency Department should be on rehydration and that only certain patients, such as those with fever or dysentery, or those with an impaired immune response should receive empiric antimicrobial therapy. More studies are needed to determine in what instances antimicrobials are of greatest benefit, so that adverse effects of rampant antibiotic prescription can be curtailed.

## BACKGROUND

Gastroenteritis (GE) is defined as the inflammation of the mucosal lining of the stomach and intestines. It is a very common presentation to the Emergency Room (ER) (and Out-Patient Departments), and the majority of cases are infectious, though a specific organism is not usually identified. According to the World Health Organization (WHO), it is a leading cause of death worldwide.^1^

Treatment is mainly supportive, with an emphasis on rehydration and correction of any electrolyte imbalances. Anti-motility drugs and anti-secretory agents may also be used. Antibiotics are not routinely indicated, and their role in management of acute gastroenteritis (AGE) is not well defined- this will be the focus of this review.

## RATIONALE

Antimicrobials can cause multiple side effects, and their inappropriate use results in development of resistance in various pathogenic organisms. Curtailing unnecessary use may result in fewer complications (and thus better treatment) as well as decrease the incident of drug resistant bacterial infections. At least one local study shows misuse/overuse of antibiotics in Pakistan.^2^

### Classification of AGE

Infectious GE can be caused by any of multiple organisms^3^ including viruses, bacteria and amoebae. As viral infection has no specific/targeted treatment, it will not be further discussed. Generally, as the causative organism will not be identified in the patient presenting to the ER with AGE, classification on the basis of the pathogen is not useful for this review. Thus, this paper will differentiate patients on the basis of presentation.

### Acute Watery Diarrhoea

This is defined as loose, watery stool (without blood, lipid, mucus, etc.) for less than 14 days without evidence of septicaemia. Acute diarrhoeal illness tends to be a self-limiting disease, so treatment is mainly supportive. Antimicrobial therapy is not routinely indicated for numerous reasons. Firstly, there is a lack of evidence that antibiotic treatment is of any significant benefit^4^-^8^, so risks associated with its prescription cannot usually be justified.

Secondly, it can result in increase in duration of fecal shedding of organisms.^8^ As a result, the infected individual can spread the infection to close contacts for a longer period of time. This can increase the burden on the health care system.

Furthermore, antibiotic administration is associated with multiple possible adverse effects, including development of *Clostridium difficile* associated colitis^9^-^12^ (*C. difficile* colitis), a disease with significant rates of morbidity and mortality. *C. difficile* colitis is more common with the cephalosporin and quinolone groups of antibiotics, as well as amoxicillin and clindamycin.

In cases of patients infected with *E. coli* strain O157:H7, there is evidence that antibiotics may precipitate development of haemolytic-uraemic syndrome (HUS), a clinical syndrome more common in children but increasingly recognized in adults, without conferring any reasonable benefit.^13^-^16^ At least one study in vitro showed that gentamycin may safely reduce the release of shiga toxin (Stx) from Shiga toxin producing *E. coli*, but evidence is lacking to recommend the use of this antibiotic in most cases.^17^ If antibiotics must be given, an animal study suggests that macrolides, such as azithromycin, may also be a safer option.^18^ However, despite the possibility of inclusion of antibiotics in the treatment regimen for *E. coli* in the future, current evidence favours the avoidance of their use wherever possible.

Finally, but no less importantly, curtailing the inappropriate use of antibiotics should prevent the development of antibiotic resistant strains. This is important at a time that multiple drug resistant strains are being frequently recognized. This is not to say that antibiotics should never be prescribed in diarrhoeal illness, however.

For example, in cases of clinically significant cholera (rice-water diarrhoea), treatment with antibiotics should be commenced^19^-^21^, as there is good evidence that this shortens the length and severity of illness.

Typhoid is another gastrointestinal (GI) infection that warrants antimicrobial therapy. Though it is classically described as causing constipation, it can present with diarrhoea in a significant percentage of patients, especially later in the disease course. Antibiotics in this case greatly alter the natural disease course and have greatly decreased the mortality caused by typhoid. *C. difficile* colitis, a possible complication of antibiotic use (as mentioned above) may require administration of metronidazole (oral/intravenous) or vancomycin (orally).^22^

Certain other groups may also benefit from antibiotic therapy: the immunocompromised (HIV, immunosuppressive drugs, recent irradiation, those with diabetes mellitus), the elderly (>65 years old), those with prostheses, those with congenital heart diseases, etc. It should be noted that there is not much in the literature regarding these groups.

### Dysentery

This is diarrhoea with blood and/or mucus. Antimicrobials should be administered in most cases of dysentery (e.g. dysenteric shigellosis)^23^-^34^ though there are caveats here, too, such as in the case of *Campylobacter* or *Enterohemorrhagic E. coli* (EHEC) infection.

In case of *Campylobacter*, there is evidence that antibiotics started more than four days after appearance of symptoms will not alter the course of the disease; earlier administration does not have established efficacy either.

*Enteroinvasive E. coli* (EIEC) infection with systemic symptoms can be treated with antibiotics but, as mentioned earlier, treatment of EHEC (especially serotype O157) infection with antibiotics is controversial.

It should be kept in mind that, in regions with poor sanitation (such as in Pakistan), protozoal infection is fairly common and can cause dysentery that will not respond to antibiotics and will require treatment with anti-protozoal drugs.

Of course, all this is assuming that the cause of bloody stools is an infective organism, as other conditions such as Inflammatory Bowel Disease (IBD) or cancers of the colorectum (CRC) can also cause bloody stools.

### Chronic Diarrhoea

This refers to diarrhoea that lasts longer than two weeks. While there is good evidence that antimicrobials are useful in management of this condition, a search should be made for non-infectious causes as well. In the ER, unless the patient appears toxic/septic, treatment should be supportive while necessary investigations are carried out, after which targeted treatment may be started, as antimicrobials would not be warranted in the myriad other conditions that can cause persistent diarrhoea (IBD, Irritable bowel syndrome, hyperthyroidism, drugs such as selective serotonin re-uptake inhibitors (SSRIs), etc.)

### Traveller’s Diarrhoea (TD)

Traveller’s diarrhoea may be defined as gastroenteritis that develops during, or shortly after, travel abroad. While TD has traditionally been treated with antibiotics^35^,^36^, some [writers] are arguing against its routine use.^37^

The reason for widespread use of short course antibiotics for management of this condition is that they have shown to modestly reduce the duration of symptoms by a few days^36^, though without much impact on disease severity. However, given that symptoms are typically mild and short lived in the first place and that risks of developing antibiotic associated diarrhoea (or other side effects, such as Stevens-Johnsons Syndrome) and causing development of antibiotic resistance strains are fairly high, routine use of antimicrobials in this scenario may not be justified and cannot be routinely recommended.^37^ Commonly used drugs in this case are ciprofloxacin or co-trimoxazole (SMX/TMP) (with ciprofloxacin apparently superior), but it is worth keeping in mind that the FDA has recently strengthened warnings on use of ciprofloxacin.^38^ Further, due to developing resistance of *Campylobacter* to fluoroquinolones in Southeast Asia, azithromycin has become the drug of choice.^39^

The World Gastroenterology Organisation recommends that antibiotics be considered for cases of TD with moderate to severe diarrhoea, or in cases presenting with fever and/or dysentery.^35^ Thus, antibiotic treatment should be limited to those with severe symptoms, or those that cannot afford to alter travel plans.

## WHICH ANTIBIOTICS SHOULD WE USE?

Antimicrobial selection depends on the suspected pathogen. For example, the drug of choice for cholera is tetracycline, though doxycycline, azithromycin, or ciprofloxacin may also be used. For typhoid, recommendations vary by geography (due to different resistance patterns). In Southeast Asia, for instance, the preferred drug is cefixime. For TD, in immunocompetent patients, the drug selected should at least cover *Shigella* spp., *Salmonella* spp., ETEC, and *Campylobacter* spp. Traditionally, ciprofloxacin and co-trimoxazole have been used, though there have been concerns recently over its side effects and development of resistance, respectively.

If the history is suggestive of amoebic infection, the recommended treatment is metronidazole and diloxanide,^23^,^40^ though there is data that suggests that a single dose of longer acting agent (e.g. secnidazole) is as effective as a course of metronidazole.^41^-^43^

Table-I summarizes the common GI pathogens and the antimicrobials that are effective in their eradication.

**Table-I:** Common GI Pathogens and Some Appropriate Treatment Options.

*Pathogen*	*Antibiotic of Choice*	*Dose*
Vibrio cholerae	Tetracycline	500mg qid (3 days)
ETEC	Ciprofloxacin	500mg bid (3-5 days)
Shigella spp. (Dysentery)	Ciprofloxacin	500mg bid (5 days)
Campylobacter spp. (Dysentery)	Azithromycin	1g qDay (1-3 days)
Salmonella (non-Typhoid; dysenteric/septic)	Ciprofloxacin	500mg bid (10-14 days)
Clostridium difficile	Metronidazole and/or Vancomycin	400mg tds (7-10 days) and/or 125mg qid (7-10 days)
Entamoeba histolytica	Metronidazole + Diloxanide furoate	750mg tds (5 days) + 500mg tds (10 days)
Giardia intestinalis	Metronidazole	400mg tds (7-10 days)

***Note:*** References for above antibiotics are found throughout the text.

## CONCLUSION

As mentioned earlier, most uncomplicated cases of AGE do not necessitate an antibiotic prescription.

In cases of clinically significant cholera or typhoid, however, antibiotics should be given.

In patients with severe illness, dysentery, signs of systemic infection, or those from high-risk groups, consider whether benefits outweigh the risks. Use patient history to guide the decision. Note that moderate dehydration in itself is not an indication of disease severity-it may instead indicate inappropriate fluid intake.

When deemed necessary, initiate therapy with an oral antimicrobial preparation if possible (i.e. if patient is tolerating oral feed; and is not septic) rather than IV administration as there is some data to suggest that IV antibiotics pose a greater risk of development of pseudomembranous colitis than their oral counterparts.

Remember that the mainstay of therapy of infectious diarrhea is to correct or prevent dehydration and electrolyte imbalances, so focus should be on administration of Oral Rehydration Salts/solution or IV fluids, not initiation of antibiotic therapy.

Future studies should focus on further defining the role of antimicrobials in the various clinical scenarios, so that an evidence-based approach may be adopted towards prescription of an antibiotic, and so that they are given only where there is a clear benefit.

## References

[1] http://www.who.int/mediacentre/factsheets/fs310/en/.

[2] Syed Rehan Ali, Shakeel >Ahmed, Heeramani Lohana (2013). Trends of Empiric Antibiotic Usage in a Secondary Care Hospital Karachi Pakistan. Int J Pediatr.

[3] Farthing MJG, Irvine EJ, Hunt RH, Infectious diarrhea (2001). Evidence-based gastroenterology.

[4] Sánchez C, García-Restoy E, Garau J, Bella F, Freixas N, Simó M (1993). Ciprofloxacin and trimethoprim-sulfamethoxazole versus placebo in acute uncomplicated Salmonella enteritis:a double-blind trial. J Infect Dis.

[5] Mandal BK, Ellis ME, Whale K (1984). Double-blind placebo-controlled trial of erythromycin in the treatment of clinical Campylobacter infection. J Antimicrob Chemotherapy.

[6] Pai CH, Gillis F, Tuomanen E, Marks MI (1984). Placebo- controlled double-blind evaluation of trimethoprim- sulfamethoxazole treatment of Yersinia enterocolitica gastroenteritis. J Pediatr.

[7] Scott DA, Edelman R (1993). Treatment of gastrointestinal infections. Ballieres Clin Gastroenterol.

[8] Nelson JD, Kusmiesz H, Jackson LH, Woodman E (1980). Treatment of salmonella gastroenteritis with ampicillin, amoxycillin, or placebo. Pediatrics.

[9] Yarinsky S, Wheeler WE (1990). Inappropriate antibiotic use and the development of Clostridium difficile colitis. W V Med J.

[10] Wiström J, Norrby SR, Myhre EB, Eriksson S, Granström G, Lagergren L (2001). Frequency of antibiotic-associated diarrhoea in 2462 antibiotic-treated hospitalized patients:a prospective study. J Antimicrob Chemother.

[11] McFarland LV (1998). Epidemiology, risk factors and treatments for antibiotic-associated diarrhea. Dig Dis.

[12] Haran JP, Hayward G, Skinner S, Merritt C, Hoaglin DC, Hibberd PL (2014). Factors influencing the development of antibiotic associated diarrhea in ED patients discharged home:risk of administering IV antibiotics. Am J Emerg Med.

[13] Wong CS, Jelacic S, Habeeb RL, Watkins SL, Tarr PI (2000). The risk of the hemolytic-uremic syndrome after antibiotic treatment of Escerichia coli O157:H7 infections. N Engl J Med.

[14] Carter AO, Borczyk AA, Carlson JA, Harvey B, Hockin JC, Karmali MA (1987). A severe outbreak of Escherichia coli O157:H7--associated hemorrhagic colitis in a nursing home. N Engl J Med.

[15] Pavia AT, Nichols CR, Green DP, Tauxe RV, Mottice S, Greene KD (1990). Hemolytic-uremic syndrome during an outbreak of Escherichia coli O157:H7 infections in institutions for mentally retarded persons:clinical and epidemiologic observations. J Pediatr.

[16] Proulx F, Turgeon JP, Delage G, Lafleur L, Chicoine L (1992). Randomized, controlled trial of antibiotic therapy for Escherichia coli O157-H7 enteritis. J Pediatr.

[17] Mohsin M, Haque A, Ali A, Sarwar Y, Bashir S, Tariq A (2010). Effects of ampicillin, gentamicin, and cefotaxime on the release of Shiga toxins from Shiga toxin-producing Escherichia coli isolated during a diarrhea episode in Faisalabad, Pakistan. Foodborne Pathog Dis.

[18] Zhang Q, Donohue-Rolfe A, Krautz-Peterson G, Sevo M, Parry N, Abeijon C (2009). Gnotobiotic piglet infection model for evaluating the safe use of antibiotics against *Escherichia coli* O157:H7 infection. J Infect Dis.

[19] Dutta D, Bhattacharya SK, Bhattacharya MK, Deb A, Deb M, Manna B (1996). Efficacy of norfloxacin and doxycycline for treatment of *Vibrio cholerae* 0139 infection. J Antimicrob Chemother.

[20] Khan WA, Bennish ML, Seas C, Khan EH, Ronan A, Dhar U (1996). Randomised controlled comparison of single-dose ciprofloxacin and doxycyline for cholera caused by *Vibrio cholerae* 01 or 0139. Lancet.

[21] Usubutun S, Agalar C, Diri C, Türkyilmaz R (1997). Single dose ciprofloxacin in cholera. Rue J Emerg Med.

[22] McFarland LV (1998). Epidemiology, risk factors and treatments for antibiotic-associated diarrhea. Dig Dis.

[23] Kelly MP, Farthing MJG, O'Grady F, Lambert HP, Finch RG (1997). Infections of the gastrointestinal tract. Antibiotic and chemotherapy.

[24] Anders BJ, Lauer BA, Paisley JW, Reller LB (1982). Double-blind placebo controlled trial of erythromycin for treatment of Campylobacter enteritis. Lancet.

[25] Bassily S, Hyams KC, el-Masry NA, Farid Z, Cross E, Bourgeois AL (1994). Short-course norfloxacin and trimethoprim-sulfamethoxazole treatment of shigellosis and salmonellosis in Egypt. Am J Trop Med Hyg.

[26] Bennish ML, Salam MA, Haider R, Barza M (1990). Therapy for shigellosis. II. Randomized, double-blind comparison of ciprofloxacin and ampicillin. J Infect Dis.

[27] Crowe M, Ashford K, Ispahani P (1996). Clinical features and antibiotic treatment of septic arthritis and osteomyelitis due to Yersinia enterocolitica. J Med Microbiol.

[28] Garcia-Laverde A, de Bonilla L (1975). Clinical trials with metronidazole in human balantidiasis. Am J Trop Med Hyg.

[29] Gayraud M, Scavizzi MR, Mollaret HH, Guillevin L, Hornstein MJ (1993). Antibiotic treatment of Yersinia enterocolitica septicemia:a retrospective review of 43 cases. Clin Infect Dis.

[30] Khan WA, Seas C, Dhar U, Salam MA, Bennish ML (1997). Treatment of shigellosis:V. Comparison of azithromycin and ciprofloxacin. A double-blind, randomized, controlled trial. Ann Intern Med.

[31] Mandal BK, Ellis ME, Dunbar EM, Whale K (1984). Double-blind placebo-controlled trial of erythromycin in the treatment of clinical campylobacter infection. J Antimicrob Chemother.

[32] Tauxe RV, Puhr ND, Wells JG, Hargrett-Bean N, Blake PA (1990). Antimicrobial resistance of Shigella isolates in the USA:the importance of international travelers. J Infect Dis.

[33] Teasley DG, Gerding DN, Olson MM, Peterson LR, Gebhard RL, Schwartz MJ (1983). Prospective randomised trial of metronidazole versus vancomycin for Clostridium-difficile-associated diarrhoea and colitis. Lancet.

[34] Williams MD, Schorling JB, Barrett LJ, Dudley SM, Orgel I, Koch WC (1989). Early treatment of Campylobacter jejuni enteritis. Antimicrob Agents Chemother.

[35] Salam SM, Advisor BG, Lindberg SP, Dite C, Republic I, Khalif RE (2012). World Gastroenterology Organisation Global Guidelines Acute diarrhea in adults and children:a global perspective.

[36] De Bruyn G, Hahn S, Borwick A (2000). Antibiotic treatment for travellers'diarrhoea. Cochrane Database Syst Rev.

[37] Hatchette BR, BinKhamis K, Mitchelmore KL, Slayter TF, Hatchette BinKhamis (2012). Antimicrobial Stewardship and the Treatment of Travelers Diarrhea:Is it Time to Re-think Recommendations?. Clini Med Rev Therap.

[38] http://www.fda.gov/Drugs/DrugSafety/ucm500143.htm.

[39] Nair D (2013). Travelers'diarrhea:Prevention, treatment, and post-trip evaluation. J Fam Pract.

[40] Vesy CJ, Peterson WL (1999). Review article:the management of giardiasis. Aliment Pharmacol Ther.

[41] Omrani G (1995). Rastegar-Lari A. Effect of Single Dose of Secnidazole in Treatment of Intestinal Amoebiasis. J Pak Med Assoc.

[42] Dupouy-Camet J (1994). Single Dose Treatments in Tropical Infectious Diarrhoea. Drug Invest.

[43] Modarresi A (2011). Comparing the Effect of Secnidazole and Metronidazole for the Treatment of Giardiasis in Children. Pediatr Res.

